# Health System Costs of a Breast Cancer Early Diagnosis Program in a Rural District of Rwanda

**DOI:** 10.1200/GO.22.46000

**Published:** 2022-05-05

**Authors:** Rashidah Nambaziira, Lysa Carolle Niteka, Jean Marie Vianney Dusengimana, John Ruhumuriza, Kayleigh Bhangdia, Jean Claude Mugunga, Marie Louise Uwineza, Vestine Rugema, Parsa Erfani, Cyprien Shyirambere, Lawrence N. Shulman, Melany Rabideau, Lydia E. Pace

**Affiliations:** ^1^ University of Global Health Equity, Kigali, Rwanda; ^2^ Partners in Health, Kigali, Rwanda; ^3^ Harvard T.H. Chan School of Public Health, Boston, MA; ^4^ Partners in Health, Kigali, Rwanda and Boston, MA; ^5^ Rwanda Ministry of Health, Kigali, Rwanda; ^6^ Harvard Medical School, Boston, MA; ^7^ Abramson Cancer Center, University of Pennsylvania, Philadelphia, PA; ^8^ Brigham and Women's Hospital, Boston, MA

## Abstract

Low-and-middle-income countries require information about effective and pragmatic strategies to facilitate earlier breast cancer diagnoses. A Breast Cancer Early Detection (BCED) program training health workers and establishing breast clinics in Burera District, Rwanda was associated with increased evaluation of breast symptoms and higher incidence of early-stage breast cancer. We examined the program's cost from the health system perspective.

**METHODS:**

The BCED program's first phase started in 2015 and included seven health centers (HCs) and Butaro Cancer Center of Excellence (BCCOE), the cancer referral center at Butaro Hospital. Patient volume and diagnoses from 2015 to 2017 were abstracted from existing databases. We retrospectively reviewed administrative data to identify start-up and ongoing operational costs. Using Time-Driven Activity-Based Costing, clinical costs per patient visit were estimated based on HC and BCCOE visits for breast health care observed in 2021.

**RESULTS:**

Over the first 2 years, there were 1,010 HC visits for breast symptoms, 210 referrals to BCCOE, and 10 breast cancers diagnosed. The BCED program's total cost was $119,511, comprising of start-up costs ($36,917), recurring operational costs ($67,711) and clinical costs ($14,883). Clinical breast assessment (CBA) at HCs cost $3.27/visit. At BCCOE, CBA-only visits cost $13.47, CBA/ultrasound cost $14.79, and CBA/ultrasound/biopsy cost $147.81. Overall, the clinical cost per breast cancer diagnosed was $1,488. The primary cost drivers were personnel at HCs (55%) and biopsy supplies at BCCOE (46%). In other districts, patients must go to their local HC and district hospital before referral to BCCOE, adding about $14.00/patient.

**CONCLUSION:**

The BCED program's cost at HCs was modest, similar to other outpatient HC services. Hospital-level care was more expensive and requiring multiple visits for biopsies increased costs. Start-up and operational costs could be reduced by using local trainers and virtual/mobile mentorship. For scalability in other districts, decentralizing ultrasound and/or biopsies to district hospitals could reduce costs.

